# Primary ossiculoplasties provide better hearing results than revisions: a retrospective cohort study

**DOI:** 10.1007/s00405-023-07835-y

**Published:** 2023-02-18

**Authors:** Judit Kálmán, Tamás Horváth, Kornél Dános, László Tamás, Gábor Polony

**Affiliations:** 1 Department of Otorhinolaryngology-Head and Neck Surgery, Bajcsy-Zsilinszky Hospital, Maglódi Út 89-91., Budapest, 1106 Hungary; 2 Department of Otorhinolaryngology-Head and Neck Surgery, Semmelweis University, Szigony Utca 36., Budapest, 1083 Hungary

**Keywords:** Titanium ossiculoplasty, TORP/PORP, Revision surgery, Ossicular chain reconstruction, Predictive factors

## Abstract

**Purpose:**

To evaluate the efficacy of ossicular chain reconstruction (OCR) in primary and revision surgeries, and to investigate the impact of the number of previous surgeries on hearing outcomes.

**Methods:**

Retrospective analysis of cases with OCR due to chronic otitis in a tertiary center between January 2018 and September 2021.

**Results:**

Altogether, 147 cases of ossicle involvement were assessed. In 91.83% (*n* = 135) OCR was performed, 96.26% of them with titanium TORP/PORP (*n* = 130), two cases with autologous prosthesis and three with piston. Mean follow-up was 8.8 months. The ABG significantly improved in the total group (TORP/PORP) from a mean (SD) of 30.94 (15.55) to 19.76 (13.36) dB (*p* < 0.0001) with 60.86% success. The best results were achieved in primary OCR with PORP implantation without cholesteatoma (89.47%). Primary cases have a significantly higher success rate in contrary to revision surgeries (72.27%, vs. 52.00%, *p* = 0.032). The only relevant predictive factor proved to be the fact of revision (*p* = 0.029). A statistically significant correlation between the number of previous surgeries and hearing results could not be proved. There was no difference in hearing outcomes between patients with only one or more than one previous surgeries in the revision groups. Neither the presence of cholesteatoma, nor the type of OCR (TOPR/PORP) and the indication of revision had an impact on postoperative ABG.

**Conclusions:**

Titanium prostheses are effective in OCR both in primary and revision cases. It is not the number of previous surgeries, but the fact of revision that influences postoperative hearing results.

## Introduction

Ossicular chain destruction is common in chronic otitis media, especially among patients with cholesteatoma. It can occur in even up to two-thirds of the cases; however, it is usually reported around 30–50% [1–4]. It is less frequent in chronic otitis media without cholesteatoma; nevertheless, it can be detected in 15–25% of these cases as well [1–4]. Incus is affected most commonly; however, stapes or seldom malleus erosion can also be found solely or in combination [2, 5, 6].

Reconstruction of the ossicular chain can be performed during tympanoplasty by using different materials. Traditionally, autologous bone was used, but artificial prostheses have become the material of choice over the past decades, especially made of titanium [7–13]. The ideal prosthesis is biocompatible, stable, easy to fit, and capable of optimal sound transmission [14]. Both autologous and titanium prostheses possess the above-mentioned characteristics with some specific advantages and disadvantages, and the choice is usually based on local institutional practices [14]. These prostheses usually by-pass the malleus, and they connect the head of the stapes with the tympanic membrane (Partial Ossicular Replacement Prosthesis—PORP), or the moving footplate with the tympanic membrane, when the stapes superstructure is also destructed (Total Ossicular Replacement Prosthesis—TORP).

Ossicular chain reconstruction (OCR) has had a long tradition and promising results for decades now. However, revision OCR cases are still challenging with more moderate hearing results to be achieved [15]. The reason behind this fact is still not crystal clear. According to Dornhoffer and Gardner, mucosal fibrosis, persistent/recurrent drainage, type of surgery (mastoidectomy/CWU/CWD), state of malleus and the fact of revision surgery have an impact on the success of OCRs, though tympanic membrane integrity or presence of cholesteatoma do not [16]. Some studies could [17], but others could not prove the role of malleal status [18] or the type of mastoid surgery [19].

In this retrospective study, we examined tympanoplasties performed in a tertiary referral center, a university ENT Department between 2018 and 2021. The object of the study was (1) to examine and compare the hearing results after primary and revision tympanoplasties with titanium PORP/TORP OCR according to indication, surgery type, and ossiculoplasty, (2) to reveal possible predictive factors of OCR related postoperative hearing gain, and finally (3) to reveal the causes of prosthesis malfunctions and the association between hearing results and the number of previous surgeries in revision OCR cases.

## Patients and methods

### Patient data

This retrospective study was approved by the Regional and Institutional Committee of Science and Research Ethics (RKEB No.: 11/2022). After approval, computerized medical charts and audiograms of all patients were reviewed who underwent tympanoplasty between January 2018 and September 2021. Patients with malignant middle or outer ear disease, stapedotomies, and tenotomies were excluded from the study. Patients who were referred to our tertiary referral center for revision OCR procedure were called as “external”, whereas patients, whose previous surgery was performed in this tertiary center were called as the “internal”. The number of patients treated in the examined period was negatively influenced by the COVID pandemic due to the temporary lock-down for elective operations.

### Surgery

All surgeries were performed by the same senior middle-ear surgeon (G.P.) with microscopic technique under general anesthesia. In cases of chronic otitis media (COM) with cholesteatoma, an attico-mastoidectomy was performed using the canal wall-up (CWU) technique whenever it was possible. Canal wall-down (CWD) mastoidectomy with canal wall reconstruction was chosen for extensive cholesteatoma cases. The transmeatal approach was primarily chosen in isolated ossicular chain disruption cases, chronic otitis media cases without cholesteatoma and attic cholesteatoma, which were completed with retroauricular incision in case of necessity. One-stage procedures were performed using titanium partial or total ossicular replacement prosthesis (PORP/TORP) (Kurz TTP-Variac System, Heinz Kurz GmbH, Dusslingen, Germany) in all patients with ossicular chain involvement, also in cases of cholesteatoma. To cover the prosthesis head, a tragal or conchal cartilage graft was used, which was previously thinned with a calibrated cartilage cutter (Kurz) to 0.4 mm. Perichondrium or cartilage-perichondrium island grafts were applied to close perforations.

Patients were followed up with diffusion-weighted MRI (DW-MRI) in selected cases of cholesteatoma that were first performed 12–18 months after surgery [20]. Second-look revision surgery was necessary only in cases when the surgeon was not convinced of the complete elimination of the disease during primary surgery, and DWI-MRI examinations could not rule out residual cholesteatoma in the middle ear.

### Hearing results

Pre- and postoperative hearing thresholds via air and bone conduction were measured, and air–bone gap (ABG) values were calculated. The audiological assessment was conducted according to the recommendation of the Committee of Hearing and Equilibrium of AAOHNS [21]. The pure tone average (PTA) threshold was determined as the mean value at 0.5–1–2–3 kHz. The very last follow-up audiogram was considered the postoperative audiogram. The difference between the pre- and postoperative ABGs was defined as “gap change”. Ossiculoplasty was considered successful at a postoperative ABG of 20 dB or less in accordance with previous studies [22]. Postoperative hearing outcomes were assessed via postoperative ABG and surgical success as well.

### Statistical analysis

Statistical analyses were performed using IBM SPSS Statistics for Macintosh v.25. (Amonk, NY:IBM Corp).

Continuous variables were expressed as mean ± standard deviation, and categorical variables as frequencies and percentage. Continuous variables were compared between the two groups with an independent samples t test or Mann–Whitney *U* test, depending on the normality of distribution of data. Of normality tests, Kolmogorov–Smirnov and Shapiro–Wilk tests were used. Chi-squared test was used to determine whether there was a correlation between groups for categorical data. The association between the number of previous surgeries and surgical success was assessed via Kendall’s tau correlation and logistic regression. Independent predictor factors of surgical success and postoperative ABGs of revision surgeries were examined with multivariate regression analysis.

A *p* value of less than 0.05 was considered to indicate statistical significance in all tests (95% confidence interval).

## Results

Altogether 330 middle ear surgeries were performed during the examined period. Seventy-seven cases were dismissed due to the exclusion criteria. Finally, 253 patients (male *n* = 143, female *n* = 110) with a mean age of 38.66 (range 3–83) met the criteria for this study. The mean postoperative follow-up time was 8.8 months (1–54).

The 253 tympanoplasties were divided into primary (*n* = 161, 63.63%) and revision surgeries (*n* = 92, 36.36%). In 100 cases the indication was COM with cholesteatoma, whereas the rest of the cases (*n* = 153) was COM without cholesteatoma or conductive hearing loss (CHL).

In 147 cases (58.10%) ossicular chain destruction was detected intraoperatively, and 135 cases (91.83%) underwent immediate ossicular chain reconstruction (OCR). Titanium prosthesis was used 130 times (96.29%) (PORP *n* = 84, 61.48%, TORP *n* = 46, 34.07%), while in three cases piston (2.22%) was required, and in two cases (1.48%) autologous prosthesis was chosen, Table 1.

**Table 1:** Clinical and demographic characteristics of the sample

	*n*	%
Overall	253	100
Gender		
Male	143	56.52
Female	110	43.47
Age (mean)	38.66 (3–83)	
Primary procedure	161	63.63
Revision procedure	92	36.36
COM w Cholest	100	39.52
COM w/o Cholest and CHL	153	60.47
Ossicular chain involvement	147	58.10
OCR	135	91.83
TORP/PORP total	130	96.29
TORP	46	34.07
PORP	84	61.48
Piston	3	2.22
Autologous prosthesis	2	1.48

*COM w Cholest* chronic otitis media with cholesteatoma, *COM w/o Cholest and CHL* chronic otitis media without cholesteatoma and conductive hearing loss cases, *OCR* ossicular chain reconstruction, *TORP* total ossicular replacement prosthesis, *PORP* partial ossicular replacement prosthesis

Altogether 92 revision tympanoplasties were performed with the indication of COM (with or without cholesteatoma, *n* = 67, 72.82%) or CHL (*n* = 25, 27.17%). In 23 cases prosthesis malfunction was revealed, which was 17.69% of all OCRs with titanium prostheses. The proportion of PORP malfunction was 15.47% (*n* = 13), whereas TORP malfunction was 21.73% (*n* = 10). The reason for malfunction turned out to be dislocation of the prosthesis (*n* = 10, 43.47%), lateralization (*n* = 1, 4.34%), insufficient length of prosthesis (*n* = 3, 13.04%), periprosthetic fibrosis (*n* = 5, 21.73%), destruction of stapes superstructure after previous OCR with PORP (*n* = 2, 8.69%), periprosthetic cholesteatoma formation (*n* = 2, 8.69%), and perforation of the eardrum (*n* = 1, 4.34%). In some cases, two different types of malformations were found at the same time.

In the group of revision tympanoplasties with prosthesis malfunction, the indication for primary surgery was COM with cholesteatoma (*n* = 18, 78.26%), COM without cholesteatoma (*n* = 1, 4.34%) or CHL (*n* = 4, 17.39%). The number of previous middle ear operations among revision cases with malfunctioning prosthesis was one (*n* = 8, 34.78%), two (*n* = 7, 28.57%), three (*n* = 4, 17.39%), four and five (*n* = 2, 8.69%), mean: 2.26. Table 2.

**Table 2:** Distribution of revision surgeries according to type of ossiculoplasty, indication, reason for prosthesis malfunction and number of previous surgeries

	*n*	%
Revision surgeries		
Total revision	92	
TORP + PORP revision	37	100
rTOPR	15/37	40.54
rPORP	22/37	59.45
Indication for revision		
COM	67/92	72.82
CHL	25/92	27.17
TORP + PORP prosthesis malfunction	23*	100
Reason for malfuncion		
Dislocation	10/23	43.47
Periprosthetic fibrosis	5/23	21.73
Insufficient length of prosthesis	3/23	13.04
Destruction of stapes superstructure	2/23	8.69
Periproshetic cholesteatoma formation	2/23	8.69
Lateralization	1/23	4.34
Perforation of eardrum	1/23	4.34
Primary indication		
COM w Chol	18/23	78.26
COM w/o Chol	1/23	4.34
CHL	4/23	17.39
Number of previous surgeries		
1	8/23	34.78
2	7/23	28.57
3	4/23	17.39
4	2/23	8.69
5	2/23	8.69
Mean	2.26	

*COM* chronic otitis media, *CHL* conductive hearing loss cases*, TORP* total ossicular replacement prosthesis*, PORP* partial ossicular replacement prosthesis, *rTORP* revision TORP*, rPORP* revision PORP, *COM w Cholest* chronic otitis media with cholesteatoma*, COM w/o Cholest* chronic otitis media without cholesteatoma, *In some cases 2 different types of malformations were found at the same time

### Hearing results and surgical techniques

Of the included 253 cases, 151 postoperative audiograms were available finally. Unfortunately, due to the COVID pandemic, a relatively great amount of patients were lost for our study during the follow-up.

Finally, the audiograms of 101 primary- and 50 revision procedures were subject to our study.

The pre- and postoperative ABGs of the patients were compared, and a significant improvement was detected overall (*n* = 151, *p* < 0.0001), in the primary (*n* = 101, *p* < 0.0001) and in the revision groups as well (*n* = 50, *p* = 0.0025). Comparing the postoperative ABGs in the primary and the revision groups, it was significantly less in the primary group (*p* = 0.032). Significant hearing improvement was detected both among COM patients with cholesteatoma (*n* = 64, *p* = 0.0009) and in patients with COM without cholesteatoma (*n* = 87, *p* < 0.0001), and the difference between these two groups with respect to postoperative ABGs was not significant (*p* = 0.912). As for primary surgery cases with cholesteatoma or without cholesteatoma, postoperative ABGs were significantly lower than preoperative results (primary with cholesteatoma: *n* = 39, *p* = 0.0082, primary without cholesteatoma: *n* = 62, *p* < 0.0001), and there was no significant difference in postoperative ABGs between the two groups (*p* = 0.084). When the hearing improvement was analyzed among revision cases with or without cholesteatoma, the hearing improvement was significant (revision surgery with cholesteatoma: *n* = 25, *p* = 0.05, revision surgery without cholesteatoma: *n* = 25, *p* = 0.0131). The achieved postoperative ABGs in the revision group with cholesteatoma were significantly lower compared to the revision group without cholesteatoma (*p* = 0.038) Table 3.

**Table 3:** Distribution of the patients according to surgery type, indication and ossiculoplasty and hearing outcome

	*n*	Preop ABG (dB) mean (SD)	Postop ABG (dB) mean (SD)	Postop ABG (dB) median	Δ ABG (dB) (SD)	Postop ABG ≤ 20 dB (%)	*p* (pre and postop mean ABG)	*p*
Overall	151	27.21 (14.82)	17.54 (12.62)	13.75	9.67 (14.62)	65.56	< 0.0001*	
Primary procedure	101	25.30 (14.54)	15.46 (10.48)	11.84	9.84 (14.69)	72.27	< 0.0001*	0.032*
Revision procedure	50	31.08 (14.77)	21.74 (15.37)	19.06	9.34 (14.63)	52	0.0025*	
COM w Cholest	64	25.84 (15.05)	17.62 (12.28)	15.62	8.22 (15.04)	62.5	0.0009*	0.0912
COM w/o Cholest	87	28.22 (14.65)	17.48 (12.93)	13.75	10.74 (14.30)	67.81	< 0.0001*	
Primary w Cholest	39	25.69 (15.19)	17.66 (10.51)	16.87	8.03 (14.56)	64.1	0.0082*	0.084
Primary w/o Cholest	62	25.05 (14.23)	14.07 (10.30)	14.07	10.98 (14.76)	77.41	< 0.0001*	
Revision w Cholest	25	26.08 (15.15)	17.55 (14.87)	10.62	8.53 (16.05)	68.18	0.05*	0.038*
Revision w/o Cholest	25	36.08 (12.80)	25.92 (15.00)	26.25	10.15 (13.33)	44	0.0 131*	
Total TORP/PORP	92	30.94 (15.55)	19.76 (13.36)	17.18	11.18 (15.90)	60.86	< 0.0001*	
TORP	33	34.60 (16.35)	23.09 (13.43)	19.37	11.52 (15.79)	51.51	0.062	0.043*
PORP	59	28.89 (14.83)	17.90 (13.07)	13.75	10.99 (16.09)	66.1	0.002*	
Primary TORP/PORP	55	29.27 (15.39)	16.97 (11.23)	15.00	12.29 (15.54)	69.1	0.006*	0.032*
Revision TORP/PORP	37	33.41 (15.56)	23.90 (15.25)	20.62	9.51 (16.48)	48.6	< 0.001*	
Primary +TORP	17	34.93 (17.85)	20.74 (9.58)	19.37	14.19 (16.29)	58.82	0.0069*	0.038*
Primary +PORP	38	26.74 (13.67)	15.30 (11.62)	11.56	11.45 (15.34)	73.68	0.0002*	
Revision +TORP	16	34.26 (15.16)	25.59 (16.55)	22.81	8.67 (15.22)	43.75	0.1328	0.565
Revision +PORP	21	32.77 (16.36)	22.62 (14.46)	19.37	10.15 (17.74)	52.38	0.0394*	
Primary w Cholest +TORP	13	31.88 (11.75)	17.98 (8.23)	18.12	13.89 (7.84)	69.23	0.0023*	0.811
Primary w Cholest +PORP	19	24.54 (13.23)	18.88 (12.88)	16.87	5.66 (13.49)	57.89	0.19	
Primary w/o Cholest +TORP	4	44.84 (9.65)	29.69 (8.85)	31.56	15.16 (10.39)	25	0.05*	Θ
Primary w/o Cholest + PORP	19	28.95 (14.11)	11.71 (9.19)	8.75	17.24 (15.19)	89.47	0.0032*	
Revision w Cholest + TORP	7	29.55 (13.05)	24.64 (14.19)	25.62	4.91 (16.52)	28.57	0.5132	0.211
Revision w Cholest +PORP	11	27.56 (19.35)	16.42 (14.96)	10.62	11.14 (19.77)	72.72	0.1465	
Revison w/o Cholest + TORP	9	37.92 (16.39)	26.32 (10.31)	18.75	11.60 (14.41)	55.55	0.0912	0.315
Revision w/o Cholest + PORP	10	38.50 (10.43)	29.43 (10.83)	30.31	9.06 (16.16)	30.00	0.0729	

*COM* chronic otitis media, *TORP* total ossicular replacement prosthesis, *PORP* partial ossicular replacement prosthesis, …*w Cholest* with cholesteatoma, *…w/o Cholest* without cholesteatoma, *preop ABG* preoperative air–bone gap, *postop ABG:* postoperative air–bone gap, *Δ ABG: gap-change, Θ* significance could not be calculated due to low number of cases, *p < *0.05, CI:95%*

#### Comparison of primary and revision OCRs

There was a significant difference between the preoperative and postoperative ABGs both in primary TORP/PORP OCRs (*n* = 55, *p* = 0.006) and in revision TORP/PORP OCRs (*n* = 37, *p* < 0.001). Hearing results with primary TORP/PORP implantation were significantly better than with revision TORP/PORP OCRs (*p* = 0.032).

#### Comparison of partial ossicular replacement and total ossicular replacement prostheses

The two groups were compared in terms of mean pre- and postoperative ABGs and gap change. As for the postoperative ABGs, postoperative ABGs were statistically lower than preoperative ABGs in PORP cases (PORP: *n* = 59, *p*  = 0.002, TORP: *n* = 33, *p* =  0.062).

Hearing results with PORP were significantly better in comparison to TORP with regard to the postoperative ABGs (*p* = 0.043) Table 3.

#### Hearing results with partial ossicular replacement prosthesis

When we evaluated the pre- and postoperative ABGs of patients who underwent surgery with PORP implantation, a significant improvement in hearing was observed both in primary (*n* = 38, *p* = 0.0002) and revision surgeries (*n* = 21, *p* = 0.0394) with PORP implantation.

Patients who had COM with cholesteatoma and underwent primary surgery with PORP implantation and patients who had COM without cholesteatoma and also had primary surgery with PORP implantation were evaluated with regard to preoperative and postoperative ABGs. The difference was significant among patients with COM without cholesteatoma (*n* = 19, *p* = 0.0032) but it was not significant among patients with COM with cholesteatoma (*n* = 19, *p* = 0.1900).

As for revision surgeries with PORP implantation among patients with COM with or without cholesteatoma, no significant difference in improvement of ABGs was observed in the two subgroups (revision surgery with cholesteatoma + PORP: *n* = 11, *p* = 0.1465 and revision surgery without cholesteatoma + PORP: *n* = 10, *p* = 0.0729) Table 3.

#### Hearing results with total ossicular replacement prosthesis

Overall, the achieved hearing improvement among patients after primary surgery with TORP reconstruction was significant (*n* = 17, *p* = 0.0069), but in revision cases with TORP this difference was not considered statistically significant (*n* = 16, *p* = 0.1328).

A significant improvement was observed in patients who underwent primary surgery due to COM with cholesteatoma (*n* = 13, *p* = 0.0023) or without cholesteatoma (*n* = 4, *p* = 0.05) reconstructed with TORP implantation.

When hearing improvement of patients who underwent revision surgery with TORP implantation for COM with or without cholesteatoma was evaluated, no significant difference could be observed (revision surgery with cholesteatoma and TORP implantation: *n* = 7, *p* = 0.5132, revision surgery without cholesteatoma and TORP implantation: *n* = 9, *p* = 0.0912). Table 3.

### Hearing results and the number of previous surgeries in revision cases

Hearing results of primary surgeries were significantly better than those of revision surgeries (*p* = 0.032), and primary TORP/PORP OCRs also had significantly lower postoperative ABGs than revision TORP/PORP OCRs (*p* = 0.032).

However, we could not prove a statistically significant correlation between the number of previous surgeries and postoperative mean ABG either in the total revision group (*n* = 50, *p* = 0.289) or in the TORP/PORP revision group (*n* = 37, *p* = 0.611) and in the prosthesis malfunctional revision group (*n* = 23, *p* = 0.307). There was no significant correlation between the number of previous surgeries and surgical success in the total revision group (*n* = 50, *p* = 0.930), in the TORP/PORP revision group (*n* = 37, *p* = 0.504) and in the prosthesis malfunctional revision group (*n* = 23, *p* = 0.780).

There was no difference in postoperative ABG between patients who had only one or more than one previous surgeries in the total revision group (*n* = 50, *p* = 0.381), in the TORP/PORP revision group (*n* = 37, *p* = 0.988) and in the prosthesis malfunctional group (*n* = 23, *p* = 0.181). As far as surgical success was concerned, no significant difference could be verified between patients who had only one or patients, who had more than one previous surgeries in the total revision group (*n* = 50, *p* = 0.555), in the TORP/PORP revision group (*n* = 37, *p* = 0.842) and in the prosthesis malfunctional group (*n* = 23, *p* = 0.297) Table 4.

**Table 4:** Hearing results and number of previous surgeries

	*n*	*p*	
		Postop ABG	Surgical success
Number of previous surgeries			
Total revision	50	0.289	0.930
TORP/POPR revision	37	0.611	0.504
TORP/PORP malfunction	23	0.295	0.78
1 versus ≥1 previous surgeries			
Total revision	50	0.381	0.555
TORP/POPR revision	37	0.988	0.842
TORP/PORP malfunction	23	0.181	0.297

*TORP* total ossicular replacement prosthesis, *PORP* partial ossicular replacement prosthesis, *postop ABG* postoperative air–bone gap, *surgical success* postop ABG ≤ 20 dB

### Hearing results and the location of previous surgeries in revision cases

OCR was successful in 65.21% (*n* = 15) of revisions among “external patients” (total *n* = 23), who did not have their primary ossiculoplasty in our tertiary center. In comparison, surgical success occurred only in 40.74% (*n* = 11) of revisions among “internal patients” (total *n* = 27); however, the difference was not significant (*p* = 0.084).

### Predictive factors of hearing outcome with TORP/PORP OCR

The type of surgery (primary or revision), the presence of cholesteatoma (COM with cholesteatoma or COM without cholesteatoma), and the type of OCR (TORP or PORP) were examined as independent predictors of postoperative ABG in TORP/PORP OCRs. Only revision versus primary procedures appeared to be significant predictors (*p* = 0.029). Neither the presence of cholesteatoma (*p* = 0.536), nor the type of OCR (*p* = 0.114) had a significant impact on postoperative ABG.

The same factors were used to detect their effect on surgical success (postoperative ABG ≤ 20 dB). None of them could be proved to be predictor factors (type of surgery *p* = 0.067, presence of cholesteatoma *p* = 0.785, type of OCR *p* = 0.262).

As for the revision TORP/PORP cases, neither the indication (*p* = 0.257), nor the presence of cholesteatoma (*p* = 0.583) and type of OCR (*p* = 0.759) could be proved to be predictive of postoperative ABG. Moreover, none of the following factors could be verified as significant predictive factors of surgical success: indication of revision (COM or CHL) (*p* = 0.587), presence of cholesteatoma (yes or no) (*p* = 0.790), and type of OCR (TORP or PORP) (*p* = 0.698) Table 5.

**Table 5:** Predictive factors of hearing outcome with TORP/PORP OCR

	*n*	Postop ABG	Surgical success
TORP/POPR total	92		
Revision (yes/no)		0.029*	0.067
Cholesteatoma (yes/no)		0.536	0.785
Type of reconstruction (TORP/PORP)		0.114	0.262
TORP/POPR revision	37		
Cholesteatoma (yes/no)		0.583	0.790
Type of reconstruction (TORP/PORP)		0.759	0.698
Indication of revision (COM/CHL)		0.257	0.587

*TORP* total ossicular replacement prosthesis, *PORP* partial ossicular replacement prosthesis, *postop ABG* postoperative air–bone gap, *surgical success* postop ABG ≤ 20 dB, *p < 0.05, CI:95%

## Discussion

In this study, ossicular chain destruction was found in 58.10% of the patients, which is similar to data in the literature [1, 2, 4]. In 91.83% of the cases, OCR was performed during primary surgery. However, 8.16% (*n* = 12) of cases with affected ossicles could not be reconstructed or there was no need to do it due to myringostapediopexy and adhesive situation (no place for PORP), intraoperatively revealed petrous apex cholesteatoma or due to recurrent cholesteatoma in patients with BAHA. Of all tympanoplasties, 36.36% were revision operations, and more than two-thirds of these were due to COM with or without cholesteatoma and a much smaller part of them due to CHL. As almost exclusively titanium PORP and TORP were used for OCR, the primary aim of the study was to examine their efficacy on postoperative hearing outcomes.

According to the literature, the success rate (postop ABG ≤ 20 dB) of OCRs ranges from 44 to 89% [9–11, 15, 23, 24]. However, some other studies showed that only 10–20% of OCRs required revision surgery at a later date [24, 25]. The present study revealed an overall (total TORP/PORP) 60.86% success-rate. With primary TORP/PORP OCRs the surgical success in our study was found to be 69.1%, in contrary to revision TORP/PORP OCRs, where we reached only 48.6% surgical success. We also found that the achievable postoperative hearing results were significantly better in primary OCRs with PORP than with TORP, which is in accordance with the literature [9–13]. The success rate ranged between 25% and 89.47% in the subgroups. The most successful OCRs were seen among patients with primary surgery with PORP implantation without cholesteatoma (89.47%, *n* = 19). According to previous studies and our data, it seems that the achievable hearing results among revision cases are poorer [15]. With respect to revision cases, the rate of success was also in favor of PORP OCRs in contrast to TORP; however, the difference was not significant. With regard to the rate of prosthesis malfunction, the occurrence turned out to be lower among PORP cases (PORP malfunction:15.47%, *n* = 13, TORP malfunction: 21.73%, *n* = 10).

This study also examined the reason for prosthesis malfunction. Extrusion was not found in this study, although it is not a common phenomenon [22, 26]. The proportion of prosthesis lateralization, periprosthetic fibrosis, destruction of stapes superstructure, periprosthetic cholesteatoma formation and perforation of the eardrum OCR was found similar to that in the literature [13, 17, 26–28].

Interestingly, the indication for primary surgery in the prosthesis malfunctional group was mainly COM with cholesteatoma, less often COM without cholesteatoma, and CHL. We also would like to emphasize that almost two-thirds (65.21%) of these cases had more than one tympanoplasties previously.

Few pre- or intraoperatively exact factors have been identified so far, which can predict the success of revision titanium OCR tympanoplasties [25]. Neither sex or age at surgery, etiology, presence of cholesteatoma, prior mastoid surgery, staged procedures, presence of tympanic membrane perforation, discharge, presence of malleus, status of stapes, nor type of ossiculoplasty (TORP vs PORP) could be proved to be predictors of success for revision titanium OCRs [25]. According to Le et al., the place of previous surgery (tertiary hospital versus other hospitals) can be predictive of postoperative hearing results, which can be explained with the presumption that more complex cases are referred to tertiary hospitals in contrast to other hospitals at lower referral levels [25]. The modified middle ear risk index is widely accepted to predict the success of primary tympanoplasties [29, 30].

Therefore, besides the primary objective of the current study to determine success rates after primary and revision OCRs with titanium prostheses, we also examined the role of cholesteatoma presence, type of surgery (primer/ revision), type of OCR (TORP/PORP) and indication for revision (COM/CHL) as possible predictive factors of surgical success and postoperative ABG in primary and revision OCRs. As for surgical success, none of the above mentioned factors could be proved to be predictors, whereas in the case of postoperative ABGs only the fact of revision was confirmed in this study as a significant predictor factor of OCR. We also examined the correlation between the number of previous surgeries and hearing results of patients with revision surgeries, particularly with prosthesis malfunction. This study revealed that in contrast to our expectations there is no association between the number of previous surgeries and postoperative ABGs either in total revision group or in TORP/PORP revision group and in prosthesis malfunctional group. Moreover, we also found that there was no significant difference in postoperative ABGs between patients who had only one, or patients who had more than one previous surgeries. Finally, in contrast to the finding of Le et al., this study could not significantly prove the role of location of previous procedures in revision OCRs [25].

To the best of our knowledge and on the basis of the literature search, nobody has so far examined the role of the number of previous surgeries in hearing results in revision OCRs, particularly in prosthesis malfunctional cases.

Our study did not reveal statistically significant differences in postoperative hearing results among patients who had only one or those who had more than one previous surgeries. Moreover, postoperative ABGs and surgical success do not correlate with the number of previous surgeries. We could only affirm that the fact of revision had an impact on postoperative ABGs. Primary OCR cases had significantly better postoperative ABG results than revision ones. However, these results should be considered with caution due to the limitation of the relatively low number of patients.

## Conclusions

The present study revealed an overall success of 60.86% in hearing rehabilitation with titanium TORP/PORP OCRs. The achieved hearing results with primary OCRs were significantly higher than with revision OCRs (69.1% vs. 48.1%). In OCRs with PORP implantation, hearing results were significantly better in primary cases, and this tendency could also be seen in revision cases in contrast to TORP implantations. Prosthesis malfunction occurred only in 17.69% of all TORP/PORP OCRs. The most common reason for malfunction turned out to be prosthesis dislocation. The fact of revision is a predictive factor of postoperative hearing results (ABG) in OCRs. The hypothesis that the number of previous surgeries had an impact on hearing results in revision cases could not be proved. More targeted studies with larger numbers of patients are needed to possibly confirm these data.

## Data Availability

Authors state that all data used in this publication are available in raw format and can be presented upon request.
